# Potent *in vitro* and *in vivo* antifungal activity of a small molecule host defense peptide mimic through a membrane-active mechanism

**DOI:** 10.1038/s41598-017-04462-6

**Published:** 2017-06-28

**Authors:** Lorenzo P. Menzel, Hossain Mobaswar Chowdhury, Jorge Adrian Masso-Silva, William Ruddick, Klaudia Falkovsky, Rafael Vorona, Andrew Malsbary, Kartikeya Cherabuddi, Lisa K. Ryan, Kristina M. DiFranco, David C. Brice, Michael J. Costanzo, Damian Weaver, Katie B. Freeman, Richard W. Scott, Gill Diamond

**Affiliations:** 1 Department of Oral Biology, University of Florida, Gainesville, FL 32610 USA; 2 Graduate School of Biomedical Sciences, New Jersey Medical School, Rutgers, Newark, NJ 07101 USA; 3 Department of Oral Biology, New Jersey Dental School, Rutgers, Newark, NJ 07101 USA; 4 Fox Chase Chemical Diversity Center, Doylestown, PA USA; 5 Division of Infectious Diseases and Global Medicine, Department of Medicine, University of Florida College of Medicine, Gainesville, FL 32610 USA; 6 Beta Pharma, Inc., Princeton, NJ 08540 USA

## Abstract

Lethal systemic fungal infections of *Candida* species are increasingly common, especially in immune compromised patients. By *in vitro* screening of small molecule mimics of naturally occurring host defense peptides (HDP), we have identified several active antifungal molecules, which also exhibited potent activity in two mouse models of oral candidiasis. Here we show that one such compound, C4, exhibits a mechanism of action that is similar to the parent HDP upon which it was designed. Specifically, its initial interaction with the anionic microbial membrane is electrostatic, as its fungicidal activity is inhibited by cations. We observed rapid membrane permeabilization to propidium iodide and ATP efflux in response to C4. Unlike the antifungal peptide histatin 5, it did not require energy-dependent transport across the membrane. Rapid membrane disruption was observed by both fluorescence and electron microscopy. The compound was highly active *in vitro* against numerous fluconazole-resistant clinical isolates of *C. albicans* and non-albicans species, and it exhibited potent, dose-dependent activity in a mouse model of invasive candidiasis, reducing kidney burden by three logs after 24 hours, and preventing mortality for up to 17 days. Together the results support the development of this class of antifungal drug to treat invasive candidiasis.

## Introduction

Infection by *Candida* species is the fourth most common nosocomial infection in the blood in the US^1^. Invasive candidiasis (IC) infections are life-threatening, and while numerous antifungals are used in treatment, including Amphotericin B, azoles and echinocandins, mortality still remains high. Mortality due to IC has been estimated to range from 15–40% for adults^2, 3^ and 19–31% for neonates and children^4, 5^. IC is frequent among very low birth weight babies (up to 20% in cases of extremely low birth weight), with a high mortality rate^6^. Fungal infection can be found in the bloodstream (candidemia), as disseminated candidiasis, or in a variety of organs, leading to endocarditis, meningitis, and endophthalmitis. Infections are predominantly due to the dimorphic fungus, *Candida albicans*. However, an increasing number of infections are due to other, non-albicans species, including *C. glabrata*, *C. parapsilosis*, *C. tropicalis*, and *C. krusei*. This shift has been at least partially attributed to an increasing use of prophylactic antifungal therapy^7^. In addition, there is an increase in resistance to these conventional antifungal therapies^8^. Even with the newest class of antifungals, the echinocandins, breakthrough IC is increasingly observed^9^. Thus, the development of new therapeutic agents that exhibit potent activity against all *Candida* species with a mechanism of action that does not lead to the rapid development of resistance is essential.

Broad-spectrum host defense peptides (HDPs) represent an evolutionarily ancient mechanism of innate immunity found in both the animal and plant kingdoms^10^. The potent activity of HDPs appears to be primarily based on their amphiphilic structure and their ability to selectively disrupt microbial membranes, possibly by channel formation^11^. This membrane active mechanism supports an important characteristic of the peptides, which is the low level of resistance development^12^. There are numerous reports describing antifungal properties of host defense peptides^13^, including activity against *Candida* and *Aspergillus* species^14, 15^. Disruption of the fungal membrane appears to be a common mechanism of action, but new modes of action involving cytoskeletal changes^16^ and other intracellular targets^17^ have been proposed for several of the HDPs. Most notably, the salivary anti-Candidal peptide histatin-5 acts by impairing mitochondrial membrane polarity in yeast^18^, and the plant antifungal peptide HsAFP-1 kills fungi by the induction of apoptosis^19^. Other notable HDPs with antifungal activity include the human cathelicidin, LL-37^20^, and the human α-defensin HNP-1^21^.

Despite their promising attributes, significant pharmaceutical issues, including poor tissue distribution, systemic toxicity, and difficulty and expense of manufacturing, have severely hampered clinical progress. Therefore, a series of non-peptidic analogues of the HDPs (HDP mimics) has recently been developed that have distinct advantages over peptides for pharmaceutical uses^22^. We have demonstrated that these mimics exhibit potent activity against both bacteria and fungi *in vitro* and *in vivo* ^23–25^. Several studies have demonstrated membrane activity for the HDP mimics against bacteria^26, 27^. However, the antifungal mechanism of these small molecules is still unknown. As with bacteria, we have observed a lack of resistance development by *C. albicans* to one such mimic^24^, suggesting that it may act on the membrane. In our initial studies, two compounds, mPE and PMX519, were identified as anti-*Candida* compounds from a very limited screen of an HDP-mimic compound library^23^. A subsequent screen of the library led to the identification of several new compounds with potent anti-fungal activity *in vitro* against both yeast and hyphal forms, low cytotoxicity to human cells, and strong *in vivo* activity in two different mouse models of oral candidiasis^25^. Here we extend these studies to elucidate the mechanism of action and to quantify the activity of one of these lead compounds, compound 4 (C4; see ref. 25 for structure). This compound kills both the yeast form and the hyphal form of *C. albicans* at the MIC within 5 minutes and 30 minutes, respectively^25^. We also demonstrate its *in vivo* activity in a mouse model of invasive candidiasis.

## Results

**Figure 1:**
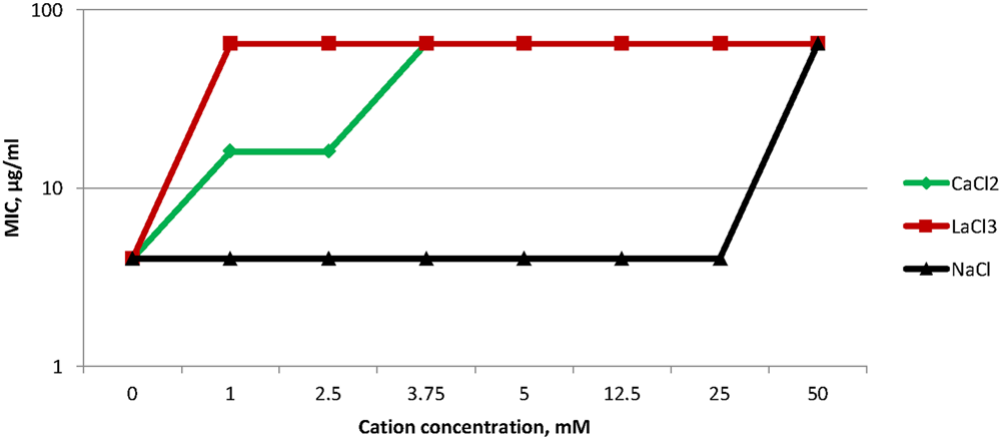
Inhibition of C4 activity by cations. MICs of *C. albicans* were determined for C4 in the presence of increasing concentrations of NaCl, CaCl_2_ or LaCl_3_. The initial MIC under standard conditions is 4 µg/ml.

It has been suggested that the initial interaction between AMPs and the microbial membrane is an electrostatic interaction between the cationic residues and the anionic headgroups found predominantly on microbial membranes^28^. To determine whether this cationic mimic has a similar initial interaction, we attempted to inhibit this interaction using monovalent, divalent and trivalent cations. Pre-treatment of the fungi with increasing concentrations of NaCl, CaCl_2_ and LaCl_3_ led to a concentration- and valence-dependent inhibition of the activity of C4 against *C*. *albicans* (Fig. 1). This suggests that, like the peptides upon which they were designed, the mimics initiate their activity by binding to the anionic microbial membrane. To determine whether there was an effect of serum components, we quantified the activity of the mimetic compound in the presence of both human and mouse serum. In 50% serum, the MIC increased only by a factor of 2, suggesting that it would still be active *in vivo* (data not shown).

**Figure 2:**
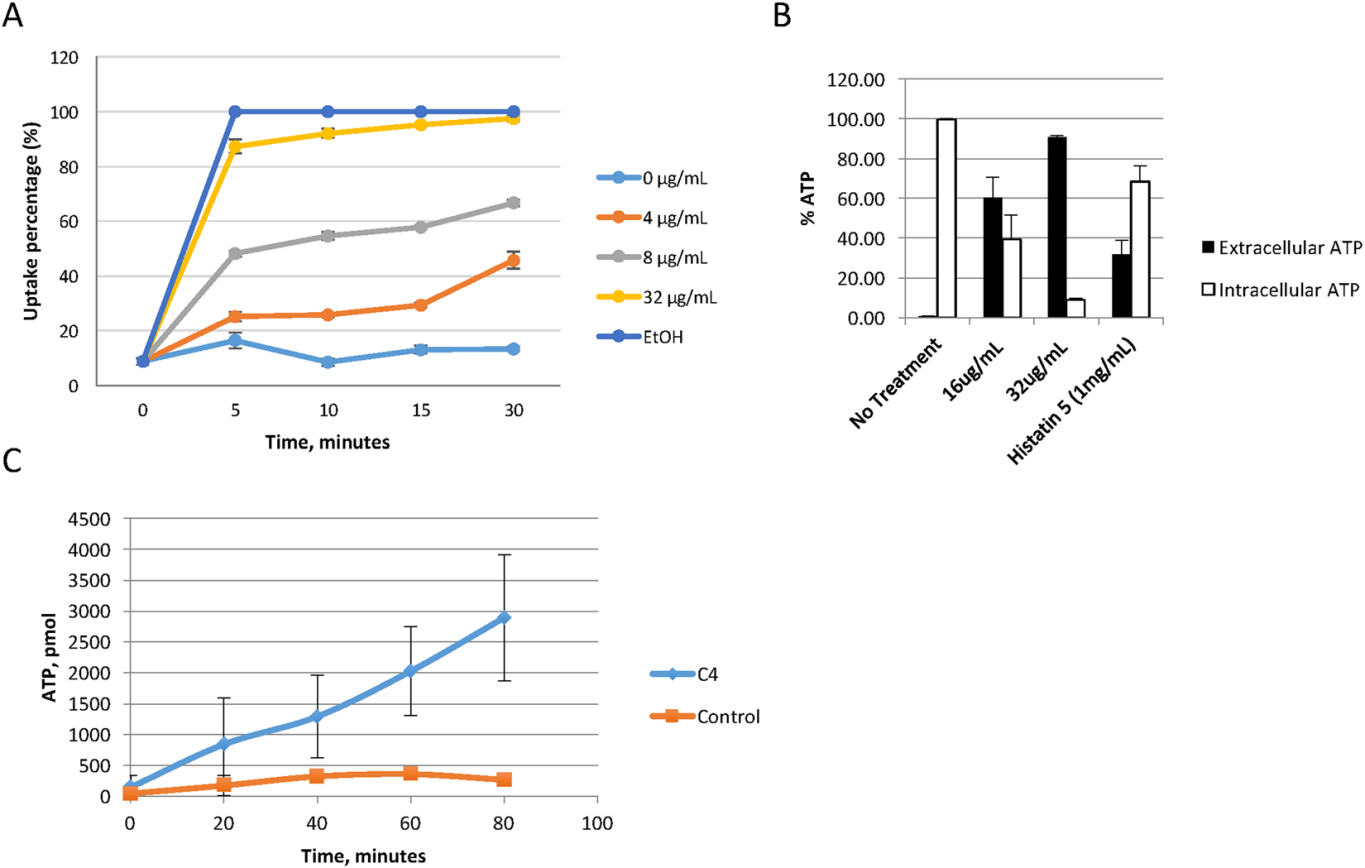
Membrane permeabilization assays. (**A**) Propidium iodide incorporation. Planktonic *C. albicans* (3.4 × 10^6^ cfu/ml) were treated with C4 at the indicated concentrations for increasing time. At each point samples were stained with PI, and incorporation was quantified by flow cytometry. 100% ethanol was used as a positive control. Shown are mean percent intracellular fluorescence of triplicate samples. Error bars indicate standard deviation. (**B**) ATP efflux in planktonic cells. *C. albicans* blastoconidia (approx. 1 × 10^7^ cfu/ml) were treated with C4 or histatin followed by quantification of intracellular and extracellular ATP. Error bars indicate standard deviation. Extracellular ATP in all treated samples was significantly greater than untreated (p < 0.01) by t-test. (**C**) ATP efflux in biofilms. *C. albicans* biofilms (n = 3) with approx. 1 × 10^7^ cfu were treated with 100 µg/ml C4, and extracellular ATP was quantified over time. Shown are mean extracullular ATP concentration +/− standard deviation.

To determine whether this HDP mimic acts at the membrane, like many HDPs, or intracellularly like histatins, the ability of C4 to cause membrane permeability was assessed. Dose-dependent membrane permeabilization of *Candida*, as shown by cellular accumulation of propidium iodide (PI), was evident within 5 minutes at 8 to 32 µg/ml concentrations (Fig. 2A). Influx was rapid, where >80% of cells were permeabilized after a 5-minute treatment with C4 at 32 µg/ml.

We similarly observed a rapid, dose-dependent efflux of ATP (Fig. 2B) in yeast cells. To determine whether this occurs in the hyphal form as well, we treated mature biofilms of *C. albicans* with the compound at 100 µg/ml and quantified the ATP efflux. As biofilms are notoriously difficult to penetrate with drugs due to the prevalence of extracellular matrix material, changes in microbe physiology, and the three-dimensional structure^29^, we were required to use a higher concentration of C4 to obtain killing of *C. albicans* biofilms^25^. These results show a time-dependent efflux of ATP from this form of *Candida* as well (Fig. 2C). Together, these data indicate that the compounds are capable of permeabilizing the membrane of *Candida* to allow the rapid influx and efflux of small molecules.

Taken together, the effects we observe on the membrane are similar to those seen with the HDPs upon which the mimic was designed^30^. Such effects are often associated with a difficulty in the development of resistance to antimicrobials^31^. To determine the ability of *C. albicans* to develop resistance to C4, we examined the effect of long-term culture of *C. albicans* in sub-MIC concentration of C4. After 30 passages at 0.5X MIC, there was no change in the MIC, in contrast to fluconazole, which developed a 60-fold increase in MIC after 16 passages (data not shown).

Some peptides, such as histatin 5, are known to act intracellularly, after energy-dependent transport across the plasma membrane, rather than on the membrane. This can be demonstrated by pre-treatment with sodium azide, which inhibits the energy-dependent membrane transport, but is not cytotoxic at low concentrations^32^. *Candida* are resistant to the activity of histatin 5 but not LL-37 or melittin when transport is inhibited^20, 33^, thus we examined the requirement for transport of C4 into *Candida* cells by examining its anti-fungal activity at the fungicidal concentration of 32 µg/ml in the presence or absence of sodium azide. We observed that while pre-treatment with sodium azide completely inhibits the cidal activity of histatin-5, there is no effect on the activity of C4, or the cytotoxic peptide melittin (data not shown). Together, the results demonstrate that there is rapid membrane activity of the mimic that does not rely on energy-dependent transport across cell membranes.

**Figure 3:**
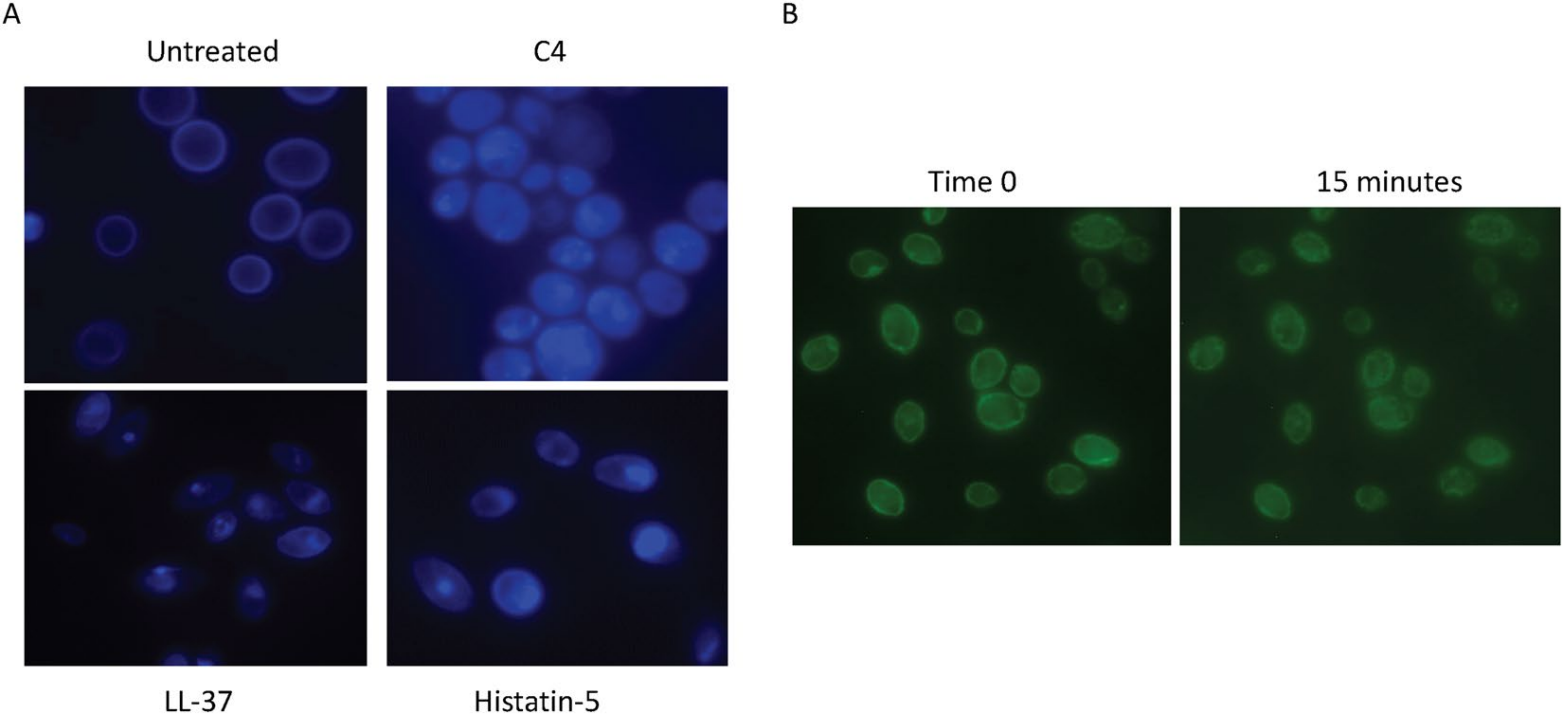
Visualization of membrane activity by fluorescence microscopy. (**A**) Filipin staining. *C. albicans* (approx. 3.4 × 10^6^ cfu/ml) were stained with filipin, and treated with the compounds listed at 32 µg/ml, and visualized under fluorescent microscopy (magnification = 1000X). Shown are representative images of Candida taken after 10 minutes. (**B**) GFP visualization *C. albicans* expressing a Pma1-GFP fusion protein in the membrane were seeded onto plates coated with concanavalin A (1 mg/ml) and treated with either histatin 5 or C4 at 32 µg/ml and visualized at time 0 and after 15 minutes.

To visualize the effect of C4 on the fungal membrane, we stained the fungi with the fluorescent drug filipin, which binds to sterols found in the *Candida* membrane. As can be seen in Fig. 3A, after a 10 minute treatment with the drug, the dye is observed to migrate into the cytoplasm suggesting a disruption of the plasma membrane, while keeping the cell wall intact. To examine this further we used a strain of *C. albicans* that expresses a membrane protein fused to GFP. By treatment of this strain of *Candida*, we can see a redistribution of the fluorescence from the membrane to the cytoplasm, further supporting the membrane activity (Fig. 3B).

To further visualize the membrane disruption we treated *C. albicans* with C4 and examined the cellular structure with electron microscopy. Consistent with our other results, using our novel fixation protocol for transmission electron microscopy (TEM), we observe effects on the plasma membrane. No effect on the cell wall was observed, in that the overall cell shape was maintained well after cell death.

**Figure 4:**
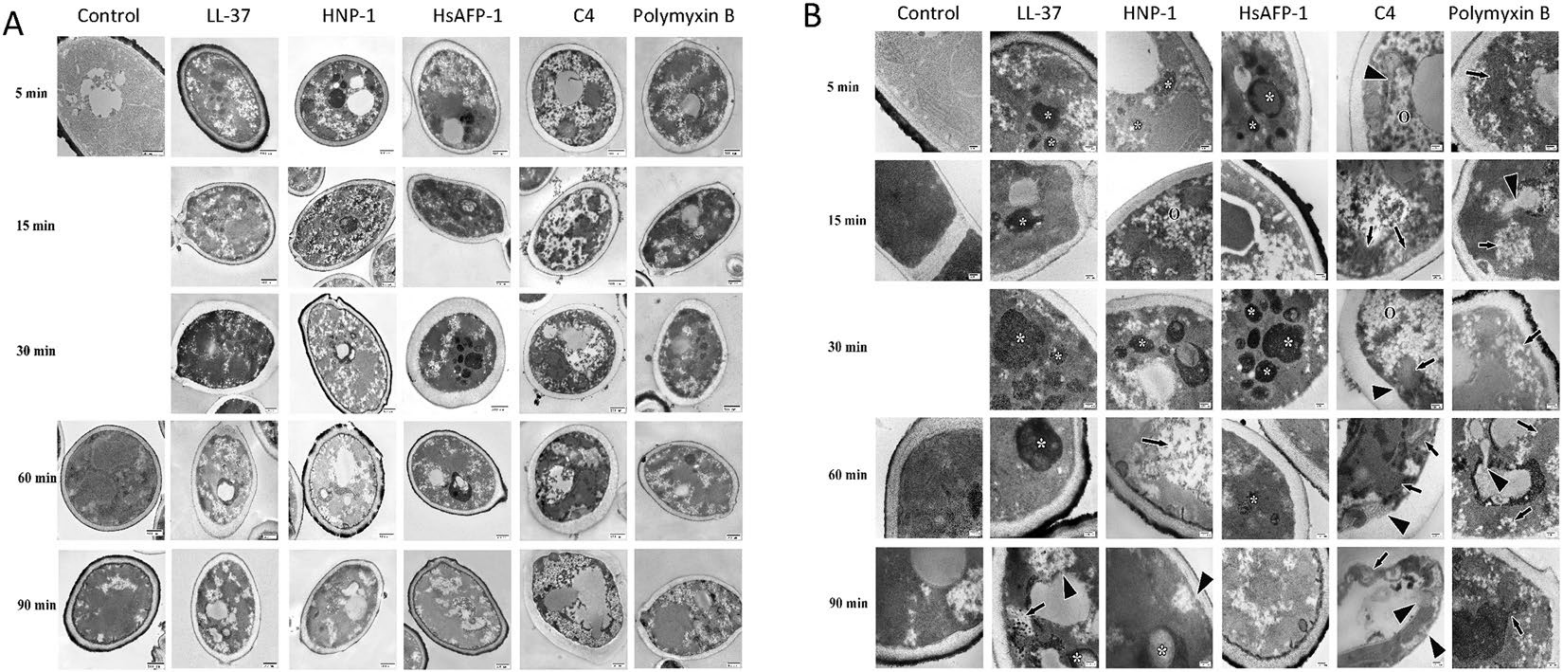
Transmission electron micrographs of *C. albicans* SC5314 treated with control or 10× the MIC of antimicrobials. *C. albicans* were treated with either PBS, C4, or the antimicrobial peptides LL-37, human neutrophil peptide (HNP)-1, *Heuchera sanguinea* antifungal peptide (HsAFP)-1 or polymyxin B. At the indicated times the yeast (3.4 × 10^6^ cfu/ml) were quickly washed, fixed and processed for embedding. Contrast was generated during post-fixing with osmium tetroxide, en bloc staining with uranyl acetate, and section staining with lead citrate. Samples were viewed at 80 kV on a Hitachi 7600. (**A**) Images were captured at a magnification of 30,000× to gain an overview of cellular changes. (**B**) Images were recorded at 100,000× magnification to see internal structural changes in the cells. The stars (*) indicate autophagy-related structures (condensed, yet fragmented nuclear fractions), large arrowheads denote clearly visible membrane ruptures and membrane internalizations (lollipops), smaller arrows are used to indicate damage to internal cell architecture, and the large ‘O’ shows internal aggregates of lipid vesicles.

Further observation using TEM demonstrates that C4 not only causes classical membrane ruptures of both the plasma membrane and of the vacuolar membrane, but also disrupts the intracellular architecture. Visible are rounded electron opaque areas, denoted by stars (*), which appear at approximately 5 minutes with C4 and with the other peptides (the cathelicidin LL-37, human defensin HNP-1, and plant defensin HsAFP-1) but not with polymyxin B (Fig. 4). By 30 minutes these structures are prominent with both peptide and C4 treatment. However, with respect to structural damage, the C4 treated series diverges from the antimicrobial peptide series by 30 minutes, and by 60 minutes the cellular deterioration in yeast treated with C4 resembles the polymyxin series, with organelle distortion (smaller arrows), membrane dissolution and internalization (large arrowheads) being observed. *Candida* treated with the peptides (LL-37, HNP-1, and HsAFP-1) appear to continue with a similar processes that are distinct from that seen with C4. The yeast treated with C4 and polymyxin for 60 and 90 minutes have grossly distorted internal structures or lack them completely, although the effects of C4 appear to be less severe than polymyxin B treatment. Polymyxin B treatment, however, does show occasional pores being formed in the plasma membrane.

We had initially demonstrated the *in vivo* effect of this compound in two mouse models of oral candidiasis^25^. To extend this analysis to systemic infections we first needed to demonstrate the *in vitro* activity against strains associated with this type of infection. We obtained clinical isolates of *C. albicans*, as well as non-albicans species (NAC), which are resistant to standard antifungal agents. The results shown in Table 1 demonstrate that C4 is active at the same level against all strains and species as with the index strain. This activity was not inhibited in the presence of 50% human serum (not shown), thus suggesting it would be active *in vivo*.

**Table 1:** Activity of C4 against disseminated candidiasis clinical isolates.

Species	Strain	Resistance	MIC (µg/ml)	MFC(µg/ml)
*C. albicans*	SC5314	none	2	8
*C. glabrata*	TG-1	fluconazole	4	16
*C. glabrata*	TG-3	fluconazole	4	8
*C. glabrata*	TG-4	fluconazole	4	8
*C. glabrata*	TG-5	fluconazole	2	8
*C. glabrata*	TG-6	fluconazole	2	8
*C. tropicalis*	CT-2	fluconazole	4	16

**Figure 5:**
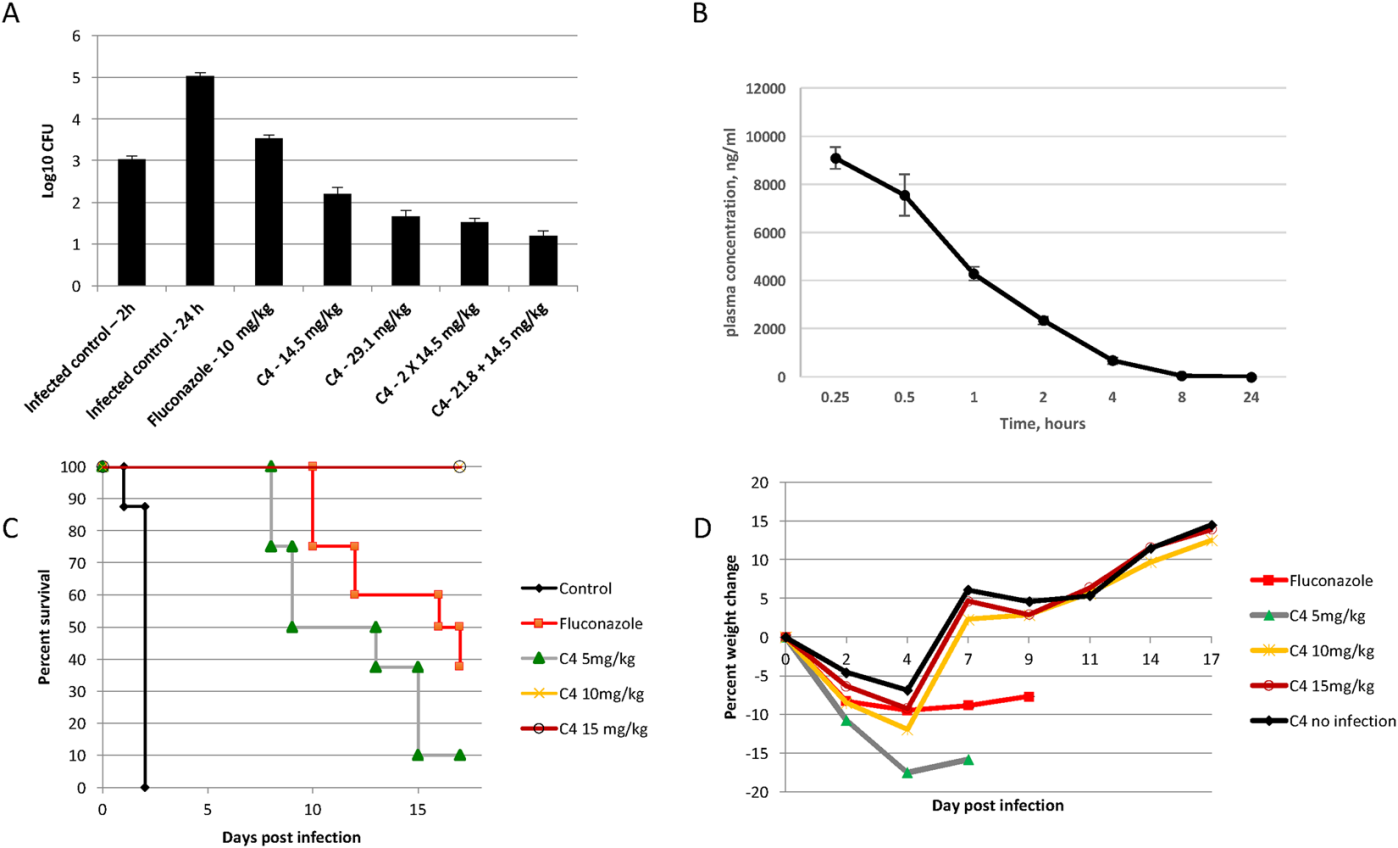
*In vivo* activity of C4. Mice were immunosuppressed with cyclophosphamide, followed by an injection of 3.4 × 10^4^ cfu *C. albicans* SC5314. (**A**) Kidney burden study. After two hours mice (n = 4) were injected with C4 at the indicated concentrations once or again after 8 hours at the indicated concentrations, or treated orally with one dose of fluconazole. Control mice were sacrificed after 2 hours or 24 hours. All other mice (n = 8) were sacrificed after 24 hours, and kidneys were removed, homogenized in PBS and plated to quantify viable cfu. Results are shown as mean +/− SEM. Differences between 24 hour control and all treated groups was significant at p < 0.001 by t-test. (**B**) Pharmacokinetics of C4 after injection. C4 was injected IV at 5 mg/kg. Blood was obtained from mice (n = 3 per sample) at the indicated timepoints, and centrifuged to obtain plasma. C4 was quantified by LC-MS/MS, and shown as mean +/− SD. (**C**) Survival study. Two hours post infection mice were injected with either PBS (control) or increasing concentrations of C4, or treated orally with fluconazole, daily for four days. Survival is shown as percent of total (n = 8 per group). (**D**) Weight change in mice from the survival study. Mean of weights of all mice were used to calculate percent weight change from time 0. In the fluconazole-treated group and the lower C4 concentration-treated group the data were censored when significant mortality was observed.

To quantify the effect of this mimic on systemic *Candida* infection, we used a standard mouse model of disseminated candidiasis^34^. Mice were immunosuppressed with injections of cyclophosphamide, followed by intravenous (IV) injection of 3.4 × 10^4^ cfu *C. albicans* strain SC5314 (originally a clinical isolate from a disseminated infection). Two hours after infection, mice were injected IV with C4 as described. After 24 hours, mice were sacrificed, kidneys were homogenized and viable colonies of *C. albicans* were quantified. Figure 5a shows that even a single dose of C4 was sufficient to reduce viable *Candida* to one log below the level seen at the time of drug administration, indicating its fungicidal activity *in vivo*. Higher concentrations and repeated treatments led to a greater reduction in kidney burden. In a second experiment (with the same immunosuppression regimen and same number of injected *Candida*), a long-term treatment was then carried out, with injection of the drug at increasing concentrations once a day for four days. Figure 5b shows the plasma concentration of C4 after IV injection at 5 mg/kg. The results in Fig. 5c show complete survival at doses above 10 mg/kg until day 17, when surviving animals were sacrificed. In addition, while an initial weight loss was observed after infection, there was a rapid increase in weight observed after the four day treatment (Fig. 5d). In contrast, the surviving mice treated with the fungistatic drug fluconazole exhibited slower weight gain, and at the end of the experiment, all surviving fluconazole-treated mice began to lose weight.

## Discussion

As systemic *Candida* infections are often fatal, and both *C. albicans* and NAC species are rapidly developing resistance to standard antifungal treatments, it is essential to develop novel antifungal agents which act via mechanisms that make it difficult to develop resistance. Our results demonstrate that the HDP mimic C4 acts in a similar way to the some of the HDPs upon which the mimics were designed, specifically with an initial electrostatic interaction with the anionic headgroups of the phospholipid membrane, followed by a rapid permeabilization of the membrane, as observed by the influx of PI and the efflux of ATP, leading to a rapid candicidal effect on both conidia and hyphae.

Most HDPs have been shown to initially interact with the plasma membrane, which in most cases leads to a permeabilization that allows rapid movement of small molecules^35^. The human cathelicidin, LL-37, for example, exhibits an azide-insensitive killing of *C. albicans*, leading to rapid PI influx and ATP efflux, supporting a similar initial mechanism for C4. This is in contrast to the salivary antifungal activity of histatin-5 which requires azide-inhibited active membrane transport to enter and subsequently kill yeast. Like C4, human defensins (HNPs), the plant defensin HsAFP-1 and other plant defensins exhibit an initial interaction with the fungal phospholipids or sphingolipids in the membrane that is inhibited by cations^13^, supporting our hypothesis that small molecule mimics of cationic HDPs target fungi through a membrane-directed pathway. However, similar to the plant defensin ApDef1^36^ we also observe some intracellular effects, suggesting multiple targets of this compound. Polymyxin B, a pore-forming antibiotic which has been used as an bactericidal agent for many years, also exhibits intracellular activity^37^. Our results show some similarities to the intracellular effects of this molecule as well. Based on this, we speculate that a combination of membrane-specific activity and intracellular activity makes it difficult for the fungus to develop resistance to C4 because of an inability to rapidly generate membrane components that do not interact with the amphiphilic compound and the HDPs.

Our results with both fluorescence microscopy and electron microscropic visualization of the membrane appear to show a further dissolution of the membrane, rather than the maintenance of pores. The pore forming antibiotic polymyxin B shows several pores, whereas C4 treated *Candida* do not. Using filipin, we observe the rapid movement of the fluorescence from the surface of the membrane to the cytoplasm after treatment with C4. This is further supported by the use of the strain of *C. albicans* expressing a GFP-tagged protein in the membrane, which shows a similar reorganization of the membrane components. This suggests a degeneration of the membrane that could occur due to the amphiphilic nature of the molecule, in a manner similar to a detergent. The membrane effects were able to be visualized by EM using a novel fixation protocol. Using this protocol we have, for the first time in yeast, used chemical fixation to show intact intracellular structure with intact glycocalyx extending from the cell walls. Previously the cell walls had to be enzymatically removed to allow acceptable preservation of intracellular structures, however the process of producing spheroblasts renders the yeast too fragile and would unnecessarily introduce ultrastructural artifacts. Using high-pressure freezing (HPF) was not economical due to the large number of samples and the replication of the experiment. The use of liposomes and other artificial membrane stuctures using lipids specific to fungi will be helpful in the further elucidation of the dynamics of the mimetic/membrane interactions in future experiments.

The subsequent steps in the fungicidal mechanism of the mimic may differ from other HDPs, which themselves also diverge^38^. Unlike the bactericidal mechanism of HDPs, which usually entails the leakage of ions and other components through pores generated by the peptides^35^, after the initial membrane interaction, fungi undergo different mechanisms of cell death. Once the initial interaction with the membrane components occurs, there is often an internalization, leading to interaction with cellular components. HsAFP-1, whose activity is sensitive to azide, leads to cell death via the generation of reactive oxygen species and ultimately apoptosis^39^. Histatins and HNP-1 are also azide sensitive, and act by a shared intracellular pathway^21^. LL-37 has been observed to affect several cellular pathways as well^40^. The recently described mechanism by which a plant defensin, ApDef1, damages fungi with the induction of endogenous reactive oxygen species^36^ is yet another example by which fungal cell death is mediated.

By TEM, we observe that treatment with C4 leads to major effects on the membrane, where sections of the plasma membrane appear to invaginate and form lollipop-shaped structures extending into the cytoplasm. Furthermore, extensive lipid microglobules were observed in the cytoplasm of some yeast, while many yeast showed round electron-dense structures and mitochondrial distortion that are found during autophagy^41^. Some of these structures are also seen with other antifungal peptides (Fig. 4), suggesting that multiple mechanisms may exist for both HDPs and the mimic. Such a drastic effect on the cell supports a two-fold fungicidal mechanism of activity – membrane disruption (both plasma and vacuolar membranes) and intracellular toxicity leading to cell death. Further studies are necessary to determine whether C4-mediated cell death is due to apoptosis, autophagy or oncosis^42^. While our data indicates that cell death by C4 in *C albicans* is primarily initiated in an autophagy-like manner we cannot exclude the possibility the C4 also induced reactive oxygen species as seen in ApDef1 treated *Saccharomyces cerevisiae*, the non-pathogenic baker’s yeast^36^. Further investigation to determine the induction of autophagy genes and the induction of reactive oxygen species may lead to a clearer interpretation of how C4 acts once inside pathogenic yeast.

One question which remains in this area is why these mimics (and the HDPs as well) are specific for microbes over the host. It has been speculated, with supporting evidence from *in vitro* studies using synthetic membranes, that the presence of cholesterol provides an inhibiting structure to the interaction of HDPs with the host membrane^43^. If this is the case, then the structural differences between cholesterol and ergosterol must be sufficient to provide sensitivity of the fungal membrane.

Our initial study of this and other mimics against *C. albicans* and NAC was performed with strains isolated from oropharyngeal candidiasis^25^. Our results here show that this mimic is equally fungicidal for clinical isolates of invasive candidiasis, even those that are resistant to fluconazole, further supporting the development of mimics as antifungal therapeutics. The observation that this mimic directly targets the plasma membrane, in contrast to the azole drugs, which target an enzyme responsible for ergosterol biosynthesis, further make it a strong candidate for its use as an antifungal agent.

The *in vivo* studies are striking in that even a single dose of C4 in a standard immunocompromised, neutropenic mouse model of invasive candidiasis was sufficient to reduce kidney burden to below the level found at the time of drug injection, a greater than 3-log reduction. This is an efficacy greater than some of the most recent echinocandins^44^. This further demonstrates that even *in vivo* C4 is acting as a fungicidal agent, rather than through a fungistatic mechanism. Further, a daily administration of either 10 or 15 mg/kg of C4 for four days was sufficient to prevent mortality in this model. Even at 17 days post infection with drug administration, there was no observable evidence of adverse reaction to C4, nor were there visible signs of persistence of the infection (not shown). To the contrary, after four days treatment there was significant weight gain in the treated animals.

Host defense peptides that exhibit potent, broad spectrum antimicrobial activity have provided the basis for significant research into the development of novel antimicrobial agents, including antifungals. Small molecule mimics represent a next generation in that development, allowing the rapid, inexpensive screening of large libraries to identify the most highly active agent with the least toxicity to the host. While a more comprehensive toxicology analysis must be performed in order to determine whether this particular mimic exhibits any acute or chronic toxic effects on the host, this study supports the further development of this class of antifungal agent as a new therapy to treat invasive candidiasis.

## Materials and Methods

### Yeast Strains

For the mechanism studies and the *in vivo* assays, we used *Candida albicans* strain SC5314, obtained from Dr. Amy Hise, Case Western Reserve University. Clinical isolates were obtained from the clinical microbiology laboratory, UF Health, and the Division of Infectious Diseases and Global Medicine, UF. *C. albicans* strain Pma1-GFP, which expresses the plasma membrane protein Pma1p fused to green fluorescent protein, was a gift from Dr. J. Konopka, Stonybrook University. Fungi are cultured on Yeast-Peptone-Dextrose (YPD) agar (1% yeast extract, 2% peptone, 2% dextrose, pH 5.7) at 37 °C. For liquid assays, single colonies were dispersed in RPMI-1640 (Mediatech, Inc.) with MOPS, pH 7.0 at a concentration of 2.5 × 10^6^ CFU/ml. MIC and MFC assays were carried out using standard NCCLS methods as we have previously described^23^. MIC and MFC determinations were determined in three independent triplicate experiments (three biological replicates with three technical replicates in each biological replicate).

### HDP-Mimic Compound

Compound C4^25^ was prepared as outlined below. For *in vitro* assays, the compound was dissolved in DMSO (Sigma) at the stock concentration of 20 mg/ml, and stored at −20 °C. Working dilutions were in RPMI-MOPS. Control experiments using vehicle alone at the diluted concentrations (never exceeding 0.0128%) showed no *in vitro* inhibitory or funcigidal effect. For the *in vivo* assays, the compound was dissolved in water and diluted to 1 mg/ml in 0.9% saline.

### Synthetic procedure for C4

#### Preparation of tert-butyl 3-(3,6-dibromo-9H-carbazol-9-yl)propylcarbamate

To a solution of 3.96 g of 3,6-dibromo-9H-carbazole and 3.01 g tert-butyl 3-bromopropylcarbamate in 30 ml of dimethylformamide was added 6.82 g of Cs_2_CO_3_ and reaction was heated to 70 °C overnight. After cooling the reaction was diluted with 100 ml ethyl acetate and washed with three 30 ml portions of water, dried with sodium sulfate and concentrated under vacuum to yield a solid. The solid was briefly warmed to 65 °C with 25 ml of ethyl acetate and allowed to slowly cool. The solvent was removed by decantation and dried to yield 2.93 g of product.

#### Preparation of 5-bromobenzene-1, 3-diol

To a solution of 100 ml of 1.0 M BBr_3_ in dichloromethane in a dried 250 ml three necked flask was added a solution of 5.40 g of 3,5-dimethoxybromobenzene in 10 ml of dry dichloromethane over 30 minutes. The reaction was allowed to stir for 16 hours at room temp. The reaction was quenched by pouring onto 400 g of ice cooled in an ice water bath. After a 30 minute age the dichloromethane was removed under vacuum and 300 ml ethyl acetate and extracted a second time with 100 ml ethyl acetate. The combined extracts were dried with sodium sulfate and concentrated under vacuum. Yield was 6.0 g of an oil with ethyl acetate present.

#### Preparation of 3-(tert-butoxycarbonylamino)propyl methanesulfonate

To an ice bath-cooled solution of 5.13 g of tert-butyl 3-hydroxypropylcarbamaten in 40 ml of dichloromethane was added 3.03 grams of triethylamine over one minute. Then 3.37 grams of methane sulfonyl chloride was added dropwise over 90 minutes. The cooling bath was removed and the reaction stirred for one hour and the dichloromethane was removed under vacuum and concentrate was partitioned between 100 ml of ethyl acetate and 50 ml of water. The organic phase was dried with sodium sulfate and concentrated under vacuum to yield 7.3 g of a liquid which was stored at −10 °C.

#### Preparation of tert-butyl 3,3′-(5-bromo-1,3-phenylene)bis(oxy)bis(propane-3,1-diyl)dicarbamate (C4)

To a solution of 3300 mg of 3,5-dihydroxybromobenzen in 25 ml of dimethylformamide was added 20.1 grams of cesium carbonate,and 6300 mg of mesylate. The reaction heated at 65 °C overnight. The reaction was incomplete and an additional 1.1 grams of mesylate and 10.0 g of Cs_2_CO_3_ were added at room temperature and the reaction was heated at 65 °C for eight hours. After stirring overnight at room temp the reaction was diluted with 200 ml of ethyl acetate and washed three times with 70 ml portions of water, dried with sodium sulfate and concentrated under vacuum to yield 7.8 g of a white solid.

### Membrane permeabilization studies

It should be noted that concentration differences between MIC (and MFC) values and concentrations used in the *in vitro* work reflect the ten-fold increase in yeast numbers required for these studies. Since, by their nature, *in vitro* studies are utilized to dissect different activities, much greater numbers of microbes are needed for reliable quantification by many *in vitro* methodologies. Biofilms are notoriously difficult for to penetrate with drugs due to the prevalence of extracellular matrix material, changes in microbe physiology, and the three-dimensional structure - all these contribute to well-documented obstruction to diffusion of compounds into biofilms^45, 46^.

1. Propidium iodide influx. *C. albicans* were grown in YPD and diluted to a concentration of 2.5 × 10^6^ CFU/ml in RPMI-MOPS. Suspensions of *Candida* were treated with mimics at the concentrations and times indicated in the text at room temperature and mixed by inversion every 30 seconds. At the indicated timepoints the yeast were washed in PBS, stained with propidium iodide (PI, 0.5 µg/ml) and subjected to flow cytometry on a Becton-Dickinson Accuri™ C6 (Becton-Dickinson, San Jose, CA) located in the Center for Immunology and Transplantation, College of Medicine, University of Florida. PI was allowed to enter yeast and bind to DNA at room temperature for 15 minutes and inversion mixing as above before being transported on ice. Ethanol treatment was used to establish 100% uptake. Experiments were repeated independently in triplicate (biological replicates), and mean percent uptake is shown. Concentrations of antimicrobials were increased concomitant to the ten-fold increase in yeast cfu to maintain approximately the same stoichiometric ratios of antifungals to yeast.
2. ATP efflux. ATP released by *C. albicans* was measured with an ATP Determination Kit (Invitrogen, Ltd.) by the luciferin/luciferase method. Briefly, planktonic solutions of *C. albicans* were standardized to an OD_595_ of 1.0 (approx. 10^7^ CFU/mL) in 1× PBS. To determine extracellular ATP release, 1 ml aliquots of this stock were incubated with the drug or PBS for 30 minutes at 37 °C, then centrifuged at 14,000 rpm for 5 minutes. The supernatant was removed and stored on ice for later measurement of ATP content. Intracellular ATP concentrations were measured as described previously^33^ with the following modifications: Cell pellets from the above experiment were re-suspended in 1 ml TE buffer to wash, then re-isolated using centrifugation. The supernatant was removed and the pellet was frozen in liquid nitrogen, followed by addition of 1 ml hot TE buffer. The samples were then boiled for an additional 5 minutes, centrifuged, and the supernatant removed and stored on ice for ATP measurement. ATP measurement was carried out according to the manufacturer instructions (Sigma). 10 µl of ATP-containing isolate was added to 90 µl of Luciferin/luciferase assay mixture in a 96-well opaque microtiter plate and light emissions were measured after 10 minutes using a BioTek Synergy HT microplate reader with KC4 software (BioTek). ATP measurements of triplicate experiments were determined from a standard curve and are reported as percent of total ATP, calculated by [ATP_extra_/(ATP_intra_ + ATP_extra_)] *100.

### Fluorescence Microscopy

*C. albicans* SC5314 were stained with filipin as described^47^. Specifically, 1 ml of an overnight culture grown in YPD (approximately 10^7^ cfu) was stained with 1 µl filipin (8 µg/ml in PBS) and incubated at room temperature for 20 minutes. The cells were centrifuged, and resuspended in PBS, and added to a chamber slide where they were allowed to settle. Antifungal drugs were added directly to the chambers and the cells were visualized by fluorescence microscopy beginning directly after addition of drugs and for the subsequent 30 minutes. Reactions of the yeast to the added drugs were generally completed within the first 10 minutes. Fluorescent images were captured with a Zeiss Axiovert 200 M microscope fitted with a Zeiss AxioCam MRm camera using a 100X (1.4 NA) objective (Carl Zeiss Microscopy, Jena, Germany) using a DAPI filter.

*C. albicans* strain Pma1-GFP were grown in YPD, and seeded onto microscope slides pre-coated with 1 mg/ml concanavalin A. Cells were visualized by fluorescence microscopy as above, using FITC filter (489 nm excitation, 511 nm emission).

Several independent replicates yielded comparable fluorescence microscopy results.

### Transmission Electron Microscopy

*C. albicans* SC5314 were grown overnight in RPMI-MOPS, harvested by centrifugation at 8,000 × g and resuspended at a concentration of 1 × 10^6^ cells/ml in RPMI-MOPS. One ml aliquots were prepared for each time point: 5, 15, 30, 60, and 90 minutes. Test and control peptides were added: 40 µg/ml C4, 80 µg/ml of plant antifungal peptide HsAFP-1 (a gift from Dr. Karin Thevissen, KU Leuven), 80 µg/ml of human alpha-defensin HNP-1 (a gift from Dr. Wuyuan Lu, University of Maryland School of Medicine), 80 µg/ml of LL-37 (GenScript), 40 µg/ml of polymyxin B (Sigma), and a non-treated control series were collected at the indicated timepoints. The concentrations of antimicrobial peptides, ten-fold higher than the MIC, were chosen to adjust for the ten-fold higher number of microbes used.

Pelleted yeast were resuspended in primary fixative (2% (v/v) glutaraldehyde (Ted Pella, Redding CA), 4% (v/v) paraformaldehyde (Ted Pella), 0.1% (v/v) DMSO (Fisher), 0.1% (w/v) Ruthenium Red (Ted Pella) in DPBS supplemented with 1 mM calcium chloride and 1 mM magnesium chloride) for 60 minutes at room temperature. After washing twice in DPBS, *Candida* were post-fixed in DPBS supplemented with 1% (w/v) Osmium tetroxide, 1% (w/v) potassium dichromate, 0.85% (w/v) sodium chloride, and 0.1% DMSO overnight at 4 °C.

Following three washes with distilled water the yeast cells were dehydrated through an extended graded ethanol series (15 min each in 50%, 70%, 90%, 95%, 100%x3) into EmBed812 (Electron Microscopy Science, Hatfield, PA). Sections were cut on a Leica Ultracut T, and stained with ethanolic uranyl acetate and lead citrate. Images were captured with a MicroFire digital camera (Optronics, Goleta, CA) and AMT Image Capture Engine (Advanced Microscopy Techniques, Woburn, MA) on a Hitachi 7600 at the Electron Microscopy Core Facility, College of Medicine, University of Florida.

### *In vivo* efficacy studies

All *in vivo* studies were carried out under a protocol approved by the University of Florida Institutional Animal Care and Use Committee, in accordance with all appropriate guidelines and regulations. Female Swiss CD.1 mice (Charles River Laboratories) were immunosuppressed by intraperitoneal cyclophosphamide injection (150 mg/kg in 10 ml/kg) on day −4 and day −1. On day 0 mice received 3.4 × 10^4^ cfu *C. albicans* SC5314 intravenously (in 0.1 ml LPS-free DPBS (Sigma) by tail vein injection) followed by either vehicle (LPS-free PBS) or C4 (at 15 mg/kg or at 30 mg/kg, in 5 ml/kg) injected IV two hours after infection. Kidneys were harvested either 2-hours post-infection or 24-hours post-infection, homogenized in 5 ml DPBS using a IKA Ultra Turrax blender, diluted in DPBS, and plated onto YPD plates. Yeast colonies were counted after overnight incubation at 37 C. Oral administration of fluconazole (20 mg/kg) was used as a positive control. Based on a power analysis (alpha = 0.5, beta = 0.20) we calculated that four animals per control group (2-hour and 24-hour DPBS), and 8 animals for each of the three drug treatment groups (fluconazole, 15 mg/kg and 30 mg/kg C4) would be sufficient. Differences were analyzed by t-test.

For the survival study, 8 animals per group (numbers arrived as per above power analysis) were treated as above with cyclophosphamide and injected with the same inoculum of *C. albicans* SC5314. Mice were treated on days 1, 2, 3 and 4 with the doses listed, and were weighed daily. Survival data was analyzed using the log-rank (Mantel-Cox) test, and one-way ANOVA with Tukey’s multiple comparisons test was performed on the change in body weight data (GraphPad InStat version 5.04).

### Quantification of C4 concentration in plasma

Compound C4 was injected into mice IV, and blood was collected. Samples were placed in tubes containing K_2_-EDTA, and then centrifuged at 8,000 rpm for 6 minutes at 4 °C and the resulting plasma were extracted with acetonitrile acidified with glacial acetic acid (9:1 v/v). Concentrations were quantified by LC-MS/MS.
